# Factors associated with cigarette smoking among learners at a high school in KwaZulu-Natal

**DOI:** 10.4102/hsag.v30i0.2832

**Published:** 2025-03-17

**Authors:** Gcinile V. Ngwenya, Refiloe M. Malaka, Lindiwe P. Cele, Modikwe Rammopo

**Affiliations:** 1 Department of Public Health, Faculty of Health Care Sciences, Sefako Makgatho Health Sciences University, Tshwane, South Africa

**Keywords:** adolescents, Amajuba district, cigarette smoking, high school learners, smoking behaviours

## Abstract

**Background:**

Young smokers are said to have increased risk of nicotine addiction, even at lower levels of consumption compared to adults, making smoking cessation among this group much more difficult. A survey previously conducted in South Africa indicated an early smoking debut age of below 18 years.

**Aim:**

This study investigated the prevalence of cigarette smoking and associated factors among high school learners.

**Setting:**

The study was conducted at a high school located in Madadeni township of KwaZulu-Natal.

**Methods:**

This cross-sectional study collected data through interviews using self-administered questionnaires. Data analysis was conducted on Epi Info 7 and STATA 17.

**Results:**

Eighty-four (21%) of the 400 participants reported smoking cigarettes. Of these, forty-nine (58.3%) had a smoking friend. Sixty out of 81 (74.1%) accessed cigarettes from the shops, whilst 69.2% (*n* = 54/78) smoked inside the school premises. Seventy-seven (92.7%) of 83 smokers expressed intention to quit smoking. Higher odds of cigarette smoking were observed among participants who were male and those who had a smoking family member, respectively, (adjusted odds ratio [AOR] = 2.33, 95% CI = 1.29–4.17) and (AOR = 5.82, 95% CI = 3.08–11.0), *p* = < 0.05.

**Conclusion:**

This study found 21% of high school learner participants who smoked cigarettes. Laws prohibiting smoking in schools and sale of cigarettes to minors should be reinforced.

**Contribution:**

intention to quit cigarette smoking as expressed by smoking learners calls for the establishment of school-based programme for smoking cessation

## Introduction

Smoking remains one of the major public health problems worldwide, with tobacco smoking responsible for more than 8 million global deaths each year (Arrazola et al. 2017). The bulk of tobacco users are reported to live in low-and middle-income countries (LMICs), contributing 80% to the 1.3 billion global tobacco users (World Health Organization [WHO] 2023). The Southeast and European regions of the WHO currently have the highest proportions of tobacco users in the population, at 26.5% and 25.3%, respectively. Women in the European region are reported to have high rates of smoking than males, which are more than double the global average (Jafari et al. 2021).

Cigarettes are the most widely used tobacco products in the world. Cigarette smoking is not harmful only to smokers, but it is also detrimental to nearby people through exposure to toxic second-hand smoke (American Lung Association [ALA] 2024b). Harmful effects include, among others, increased risk of latent tuberculosis (TB) progressing to active TB and poor prognosis, increased risk of chronic obstructive pulmonary disease (COPD), lung cancer, diabetes, premature babies, reproductive health in women, low birth weight babies and stroke among others (National Health Services 2022). Adolescent cigarette smoking has been linked with severe respiratory illness, lung growth and function, and reduced physical fitness (ALA 2024a).

Available evidence suggests an increased risk of nicotine addiction among young smokers, even at lower levels of consumption compared to adult smokers, making smoking cessation among this group much more difficult (Mahajan, Homish & Quisenberry 2021). Nicotine is known to elicit serious changes in parts of the developing teenage brain, some of which are involved with emotion regulation (Castro, Lotfipour & Leslie 2023). Studies have reported an association of nicotine dependence with smoking debut age of younger than 20 years. Surveys conducted among school learners in South Africa reported smoking at the debut age of 13 years old. This corroborates reports of tobacco and nicotine use by children aged between 13 and 16 years old (WHO 2024).

Among the studies that have investigated the factors associated with cigarette smoking, having a smoking friend or family member has been found to stimulate the initiation of smoking (Asif et al. 2017). The influence of advertisements of tobacco products on television, movies and magazines and the lack of awareness and knowledge among the learners have also been cited (CDC 2023; Sun et al. 2022). Others have also cited depression, anxiety and stress as predictors of cigarettes among school children (Fluharty et al. 2016). This study aimed to determine the prevalence of cigarette smoking and investigate associated factors among high school learners.

## Research methods and design

### Study design

This study used a quantitative, descriptive, cross-sectional design and conducted interviews using self-administered questionnaires among the learners in one of the high schools in Amajuba District in KwaZulu-Natal (KZN). The collected data were used to determine the prevalence of cigarette smoking and the associated factors.

### Study setting

This study was conducted in one of 12 high schools situated at 1014 Madadeni, Newcastle, 2957 of Amajuba district in KZN province, South Africa. Amajuba borders the Free State and Mpumalanga provinces. It is a public secondary school classified as Quintile 3 that caters to a community with moderate socio-economic status. The school has a total of 1816 learners and 56 educators, resulting in a student-teacher ratio of 32:1. The school operates from 07:20 a.m. to 14:30 a.m. (SchoolsDigest 2024).

### Data sources and target population

The study used a self-administered questionnaire written in English and translated into isiZulu, the most spoken language in the area. The data collection tool comprised of three sections. Section A collected the sociodemographic data, including the learner’s age, sex and grade. Section B collected question items to capture smoking behaviour and influences of smoking. Section C collected the reasons for smoking among the participants who had indicated that they smoked cigarettes.

The study population were all learners attending one of the high schools of Madadeni in the Amajuba district. The learners were eligible for the study if they were 12 years old and above. Potential study participants were excluded from participating if they did not offer signed assent or parental informed consent forms and if they were writing examinations during data collection. The data collection occurred between August 2022 and October 2022

### Sample size and sampling technique

The authors used the Raosoft sample size calculator (Raosoft 2004) to determine the recommended sample size. The total headcount of 1800 learners was used for the population size, and the authors selected a confidence level of 95%, which corresponds to a 5% margin of error, and chose a 50% response rate to obtain a minimum recommended sample size of 317 participants. However, because the study used self-administered questionnaires, a 25% buffer was added to maximise the response rate to obtain a final sample size of 396, which was increased to 400.

A line list of learners was arranged according to learner grade, and proportionate sample sizes of each grade were determined. For the selection of participants, the list of learners was arranged alphabetically by name before using the systematic random sampling technique, where every *n*th learner was selected. The *n* value was obtainable by dividing the total number of learners in each stratum by the required number of participants from each stratum.

### Recruitment

The recruitment of participants started immediately after permission was granted by the KZN Department of Education. The researcher made an appointment with the principal to discuss the study and the logistics regarding access to the learners. The study was explained to the selected learners, and they were given informed consent to give to their parents to sign and return them to the school. Those who returned signed forms were recruited during breaks and in the afternoon. One classroom was allocated to the researcher, where a group of students assembled to answer the self-administered questionnaires.

### Variables

The outcome variable was defined based on whether the participants had reported that they smoked cigarettes or not (smoking ‘yes’ and smoking ‘no’). The prevalence of smoking was calculated as the proportion of participants who were smoking out of the total number of the study participants. To investigate the factors associated with cigarette smoking, the smoking status variable was dichotomised into ‘yes’ and ‘no’ categories and used as the outcome or dependent variable in the regression analyses. Sociodemographic characteristics and the data related to smoking behaviours and influences of smoking were used as the explanatory (independent) variables.

### Data analysis

The study used STATA version 17 software for the analysis of data. The authors described the sample using means and the standard deviations (s.d.) for continuous data and presented the categorical data as proportions and percentages. The Chi-square test was used in the cross-tabulation of the outcome variable, cigarette smoking status and the other independent variables, including the sociodemographic characteristics and information on smoking behaviours and influences of smoking. *P* values were used to indicate the statistical significance of the differences, *p* < 0.05. Univariable and multivariable logistic regression analyses were conducted to investigate the factors associated with smoking cigarettes. All variables yielding *p* values ≤ 0.20 from the binary regression analysis were included in multivariable regression analysis. The results were reported as adjusted odds ratios (AOR), with the corresponding 95% CIs. Associations yielding *p* values < 0.05 were considered statistically significant.

### Validity and reliability

The question items of the data collection tool were adapted from previous studies. The tool was piloted on 5% of the required sample size from the same school before data collection. However, the pilot results were not included in the results of the main study. Minor modifications were made where necessary based on the participant responses.

### Ethical considerations

This study obtained ethical approval from the research ethics committee of Sefako Makgatho University Health Sciences University (SMUREC/H/42/2022:PG). Permission to conduct the study was obtained from the KZN Department of Education (Ref:2/4/8/7322). All selected learners were notified about the study and were requested to give written informed consent through the signing of the informed consent form (ICF) and assent forms. The assent forms were signed by the parents or guardians of the learners who were aged below 18 years.

## Results

### Sociodemographic characteristics of the study participants

Table 1 shows the demographic characteristics of the 400 study participants. The majority, 54% (*n* = 225) were female. The mean age of the participants was 16 years and two standard deviations (± 2 [s.d.]), with 92.8% (371) participants who were between the ages of 12 and 18 years old. More than half, 51.8% (*n* = 207) were in Grades 9 and 11. Some, 57.3% (*n* = 227) were Christian. Additionally, 75.8% (*n* = 310) lived in semi-urban areas. Cigarette smoking was reported by 21% (*n* = 84) of the participants. The mean age at which participants started smoking was 14.8 years and 1.79 s.d. (14.8 ± 1.79).

**TABLE 1:** Sociodemographic characteristics of participants, *N* = 400.

Variables	Frequency	Percentage	Mean	s.d.
**Sex**				
Female	216	54.0	-	-
Male	184	46.0	-	-
Age	-	-	16	2.1
**Age groups (years)**				
12–15	175	43.8	-	-
16–18	196	49.0	-	-
≥ 19	29	7.2	-	-
**Learner grade**				
8	74	18.5	-	-
9	84	21.0	-	-
10	107	26.8	-	-
11	92	23.0	-	-
12	43	10.8	-	-
**Religion, *n* = 396**				
Other	169	42.7	-	-
Christian	227	57.3	-	-
**Geographic location, *n* = 397**				
Rural	96	24.2	-	-
Semi-urban	301	75.8	-	-
**Smoking status**				
Yes	84	21	-	-
No	316	79	-	-
Age at smoking debut, *n* = 83	**-**	-	14.8	1.79

s.d., standard deviation.

### Reasons for smoking cigarettes

**FIGURE 1:**
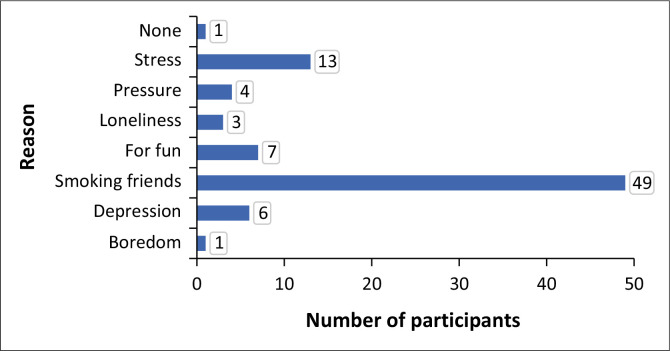
Reasons for smoking cigarettes, *N* = 84.

Figure 1 reveals that 58% (49/84) of participants smoked cigarettes because they had a friend who was smoking. Other reasons for smoking include stress, 15.5% (13), 8.3% (*n* = 7) smoked for fun, 4.8% (*n* = 4) because of peer pressure, 3.6% (*n* = 3) had loneliness, 1.2% (*n* = 1) because of boredom and 1.2% (*n* = 1) did not state the reason for smoking cigarettes.

### Smoking behaviours and influences of cigarette smoking

Table 2 shows the smoking behaviours and influences of smoking among the 84 cigarette smokers. Sixty per cent (50/83) had started smoking when they were between the ages of 12 and 15 years old, and these were mostly males, 61.0% vs. 59.4% female participants. Sixty-seven per cent (56/83) of participants were daily smokers, and there was no statistically significant difference between male and female participants, 39.3% female vs. 61% male participants, *p* = 0.69. A total of 74.1% (60/81) participants got the cigarettes from the shops, and these were mostly males, 60% vs. 40% female participants, *p* = 0.59. Of the 54/78 participants, 69.2% smoked in school, 40.7 % female vs. 59.3% male participants, *p* = 0.54. Ninety per cent (75/83) had a smoking friend, 38.7% female vs. 61.3% male participants, *p* = 1.00. Of the 66/84 smokers, 78.6% had a smoker in the family, and these were mostly male participants, 60.6% vs. 39.4% females, *p* = 0.64. Only 25% (21/84) belonged to a social club, and these were male participants, 81% vs. 19% females, statistically significant, *p* = 0.04. Ninety-three per cent (77/83) of the participants indicated an intention to quit smoking, 39% female vs. 61% male, *p* = 1.0.

**TABLE 2:** Smoking behaviours and influences of cigarette smoking, *N* = 84.

Variable	Total		Female, *n* = 32		Male, *n* = 52		*p*
	*n*	%	*n*	%	*n*	%	
**Age at smoking debut (years), *n* = 83**							
10–12	6	7.0	3	50	3	50	0.83
12–15	50	60.0	19	38	31	62.0	-
16–19	27	33.0	10	37	17	63	-
**Frequency of smoking, *n* = 83**							
Daily	56	67.4	22	39.3	34	60.7	0.63
Weekends only	13	15.7	6	46.2	7	53.8	-
Weekly	14	16.9	4	28.6	10	71.4	-
**The place where cigarettes are accessed, *n* = 81**							
School	21	25.9	7	33.3	14	66.7	0.59
Shops	60	74.1	24	40	36	60	-
**Smoking in school, *n* = 78**							
No	24	30.8	8	33.3	16	33.3	0.54
Yes	54	69.2	22	40.7	32	59.3	-
**Smoking friend, *n* = 83**							
No	8	9.6	3	37.5	5	62.5	1.00
Yes	75	90.4	29	38.7	46	61.3	-
**Smoker in the family**							
No	18	21.4	6	33.3	12	66.7	0.64
Yes	66	78.6	26	39.4	40	60.6	-
**Belongs to a social club**							
No	63	75.0	28	44.4	35	55.6	0.04
Yes	21	25.0	4	19	17	81	-
**Intention to quit, *n* = 83**							
No	6	7.0	2	33.3	4	66.7	1.00
Yes	77	93.0	30	39	47	61	-

### Factors associated with cigarette smoking

Table 3 shows the results from the multivariate logistic regression analysis. Variables of sex, having a smoking family member, age of participants and religion had statistically significant associations with cigarette smoking. The odds of cigarette smoking were higher among males compared to females (AOR = 2.33; 95% CI: 1.29–4.17), *p* = 0.004, and among participants who had a smoking family member compared to those who did not have a smoking family member (AOR = 5.82; 95% CI: 3.08–11.0), *p* < 0.001. Family in the context of this study refers to parents, siblings, aunts, uncles and cousins. Lower odds of cigarette smoking were observed among participants who were aged between 12 and 15 years old (AOR = 0.04; 95% CI: 0.01–0.15), *p* < 0.001 compared to those who were aged ≥ 19 years old and among participants belonging to the Christian religion compared to those of other religions (AOR = 0.01;95% CI: 0.00–0.18), *p* = 0.002.

**TABLE 3:** Factors associated with cigarette smoking.

Characteristic	Unadjusted		*p*	Adjusted		*p*
	OR	95% CI		OR	95% CI	
**Sex**						
Male	2.26	1.38–3.71	0.001*	2.33	1.29–4.17	0.004*
Female	-	1	-	-	-	-
**Age group (years)**						
12–15	0.08	0.03–0.21	< 0.001*	0.04	0.01–0.15	< 0.001*
16–19	0.54	0.25–1.19	0.13	0.36	0.13–0.18	0.05
≥ 19	-	1	-	-	1	-
**Learner grade**						
≤ 10	0.47	0.29–0.77	0.002*	1.64	0.85–3.18	0.14
> 10	-	1	-	-	1	-
**Religion**						
Christian	0.05	0.00–0.48	0.01*	0.01	0.00–0.18	0.002*
Other	-	1	-	-	-	-
**Geographic location**						
Semi-urban	0.15	0.01–1.63	0.05	-	-	-
Rural	-	1	-	-	-	-
**Smoker in family**						
Yes	5.68	3.21–10.01	< 0.001*	5.82	3.08–11.0	< 0.001*
No	-	1	-	-	1	-
**Smoking friend**						
Yes	1.15	0.00–0.56	0.92	-	-	-
No	-	1	-	-	-	-
**Belongs to a social club**						
Yes	0.85	0.49–1.48	0.56	-	-	-
No	-	1	-	-	-	-

CI, Confidence interval; OR, odds ratio.

* Statistically significant.

## Discussion

Our study results on the prevalence and frequency of cigarette smoking were found to be comparable to the results reported from previous surveys conducted among adolescents and young adults in South Africa. These include a systematic review study of the prevalence of tobacco use among adolescents and young adults in South Africa, which found a pooled prevalence of tobacco use of 22% (Londani & Oladimeji 2024) and Zwane, Mashau and Moselakgomo (2022) found a higher percentage of 64.7% of learners who smoke cigarettes in the school premises. However, much lower and higher smoking prevalence of 4% and 33% have been reported, respectively, among high school and university students (Roble et al. 2021; Tezera & Endalamaw 2019). Surveys carried out in America also show a high prevalence of smoking among high school learners. In 2019, Wang found that 53.3% of high school learners and 24.3% of middle school learners reported that they had tried cigarette smoking. In 2022, a survey conducted by Gentzke found a decline of 34% in cigarette smoking among high school students and 11.3% of middle school students.

Participants in this study smoked cigarettes mostly because they had a smoking friend and were stressed. Similarly, others have reported a high likelihood of smoking among high school and university students because of smoking friends and peer pressure (Desai et al. 2019; Leshargie et al. 2019). Stress alleviation has also been cited as a reason for smoking among students at a large midwestern university (Nichter, Nichter & Carkoglu 2007). This finding of more than half of daily smokers among the smoking participants has been observed in other studies. These include a study that reported 51.2% of daily smokers. Others have, however, reported daily smoking among only 5.9% of the participants (Sun et al. 2022; Wamamili et al. 2019). This study also found that some of the smokers accessed cigarettes from the shops, while others used the cigarettes while at school. A study that was conducted among adolescents in India found school learners who purchased cigarettes from a shop nearby the school (Kedar & Gupta 2021).

This study investigated the factors that were associated with cigarette smoking and found high odds of cigarette smoking among the male participants. Those who had a smoking family member have been reported in other studies. These include a study which was conducted among high school students in Eastern Ethiopia, which reported 4.33 times higher odds among male learners compared to females (Roble et al. 2021).Another is a study which was conducted in Saudi Arabia, which found an increased likelihood of tobacco use among students who had at least one member of their family who smoked (Al-Makadma et al. 2015; Reda et al. 2012). However, others have reported no significant association between living with people who smoke cigarettes and adolescent smoking (Reda et al. 2012). In this study, the authors found stress, peer pressure and having fun, among other reasons for smoking. The study by Araújo (2019) resonates with our findings that pleasure, relaxation and manipulation were recorded among reasons for adolescents smoking. A significant proportion of participants who smoke expressed a desire to quit smoking in this study, especially males. Whereas in other studies, a higher percentage of both males and females were found to have difficulties in quitting smoking, with almost a quarter of smokers intending to quit (Marques-Vidal et al. 2011). Low odds of smoking were observed in this study among adolescents who were Christians or who belonged to other religious groups, and this is comparable with the study by Alexander et al. (2016), which found that black adolescents who had a stronger religious belief prevented them from smoking, which means the odds of smoking were low among this group.

## Limitations

This study had limitations, these incudes a small sample size, which could potentially affect the statistical significance of some of the observed relationships. Since the data collected by this study was self reported, it could have introduced social desirability biases in smoking behaviours, potentially leading to an underestimation of the magnitude of the prevalence of cigarette smoking.

## Conclusion and recommendations

The finding that the average smoking debut age among school learners is 14 years necessitates urgent national attention. South Africa has proposed a bill that would impose fines or sentences of up to 15 years in prison for anyone or any business that sells cigarettes to children under 18 years old. This bill aims to align with the WHO Framework Convention on Tobacco Control (FCTC) and could help reduce the sale of cigarettes to underage schoolchildren. The prevalence of cigarette smoking in schools indicates a significant gap in the enforcement of strict rules, which must be addressed. Additionally, the intent to quit smoking expressed by some of the smoking learners calls for the establishment of a school-based smoking cessation programme. This programme should be integrated into life orientation subjects, where students are taught about the dangers of smoking.
